# Standardization of Exchanged Water with Different Properties in China’s Water Rights Trading

**DOI:** 10.3390/ijerph17051730

**Published:** 2020-03-06

**Authors:** Junyuan Shen, Fengping Wu, Qianwen Yu, Zhaofang Zhang, Lina Zhang, Min Zhu, Zhou Fang

**Affiliations:** 1 Business School, Hohai University, Nanjing 211100, China; 2 National Engineering Research Center of Water Resources Efficient Utilization and Engineering Safety, Nanjing 210098, China; 3 Business School, Suzhou University of Science and Technology, Suzhou 215009, China; 4 Department of System Designing Engineering, University of Waterloo, Waterloo, ON N2L 3G1, Canada; 5 College of Economic and Management, China Three Gorges University, Yichang 443002, China; 6 Business Administration School, Hohai University, Changzhou 213022, China

**Keywords:** water rights trading, standard water (SW), rewarding excellence and punishing inferiority

## Abstract

Water rights trading is an effective way to optimize the allocation of water resources. However, the existing practice of water rights trading in China lacks any consideration of the practical value of the exchanged water. This deficiency may lead to disputes between transferor and transferee during the implementation of the water rights trading contract. This paper puts forward the concept of Standard Water (SW). First, getting the original value of exchanged water by the shadow price model based on input-output table; Second, based on the original value, building the economic profits or costs model to obtain the practical value of exchanged water; Third, establishing SW quantity measurement model according to the principle of rewarding excellence and punishing inferiority, so as to convert the water quantity of exchanged water into SW quantity. With the standardization method, this paper takes the water rights transaction between Dongyang City and Yiwu City in 2000 as an example to carry out case study, and provides policy recommendations. The results show that when the contract requires the provision of 49.999 million m^3^ water of Class I the quality, if the exchanged water quality provided is in Class II-V, the corresponding SW will be decreased to 48.699–37.399 million m^3^. The application of this research will be conducive to ensuring the fairness and durability of the water rights trading processes.

## 1. Introduction

The global demand for water resources keeps increasing and water pollution problems will continue to worsen [1]. In order to cope with the increasingly severe water crisis, the Chinese government has actively advocated the construction of a water rights system [2,3,4,5], and achieved remarkable results. In 2000, the water rights trade between Dongyang City and Yiwu City became the first such practice in China. In the following decade, many areas have explored water rights trading in various forms. Water rights trading in China nowadays presents two characteristics: (1) The transaction contracts are usually long-term contracts (many of the execution periods are 25 years), or even a permanent contract. (2) Although the water quantity and water quality are specified in the contract, the transferor often pays more attention to the commitment of water quantity in the process of implementation. In this way, dissatisfaction, or even water disputes may occur if the quality or other properties of the exchanged water change during the execution process. Therefore, it is necessary to measure the changing value of water in the transactions and convert the water with different values using a certain standard [6].

Water rights trading is a complex system, which involves many influencing factors such as water resources endowment, water quality and so on. The regional differences of these factors make the value of water resources in different regions unequal [7]. For the water rights transferee, how to evaluate the practical value of exchanged water to its economic and social development is the core issue of SW conversion. Some scholars have studied the evaluation of water resources value, providing a reference for the SW measurement in this paper. Shen et al. [8] defined the connotation of water resources value from different perspectives such as land rent theory, labor value theory and marginal utility theory. Wei et al. [9] studied the evolution of the social value of Australian water resources for economic development and environmental sustainability from 1843 to 2011. Gao et al. [10] used matter element analysis and substitution market method to establish water resources value model, and analyzed water quality and water quantity, natural population growth rate, per capita GDP and other indicators. Jia et al. [11] and Lin et al. [12] applied the fuzzy optimization method to the value accounting of water resources. However, the shadow price method is still the most widely used method in water resources valuation [13,14,15]. For example, Zhu et al. [16] calculated the shadow price of water resources in Huaihe River Basin, and obtained the theoretical water resources value of different water use sectors in each river section. Mao et al. [17] also used the shadow price method to calculate the theoretical value of water resources in the Yellow River. Compared with other evaluation methods, the shadow price of water resources can reasonably reflect the marginal contribution of per unit water to social and economic development after optimal allocation. Therefore, this paper uses the shadow price method to reflect the marginal contribution of per unit water resources, and it is defined as the original value of the exchanged water.

In addition to the original value of water, water quality is an indispensable factor when water rights are traded as commodities. If the water quality of the exchanged water does not meet the standard, it will easily lead to transaction conflicts between the transferor and the transferee. At present, water quality has been considered in most water rights management studies. Manshadi et al. [18] considered that inter-basin water diversion should meet the water quality requirements, and proposed a water quality evaluation method for inter-basin water diversion project based on the concepts of cooperative game and virtual water. Weber et al. [19] discussed the water intake management of drinking water reservoirs, considering minimizing the environmental impact while ensuring the quantity and quality of drinking water in the reservoirs. Wang et al. [20] studied the allocation of water resources based on water quantity, water quality, environmental and other factors in the river basin, and established the water quantity-water quality-environment coupling allocation model. Cheng et al. [21] considered the influence of water quality factors in regional water resources pressure assessment, and proposed a hot spot quantitative analysis method based on water quantity and water quality. Aalami et al. [22] considered the impact of water quality damage on reservoirs, and managed water quality and quantity of reservoirs sustainably through reservoir operation strategy and watershed control strategy. Wu et al. [23] constructed a water quantity-quality coupled model for the provincial initial water rights allocation. In following studies, Min et al. [24,25] designed an initial water rights allocation model including total water consumption, water use efficiency, water quality functional area, regional coordination and sharing. Cazcarro et al. [26], and Dilekli and Cazcarro [27] extended the World Trade Model by creating water treatment sectors and provide alternative sources of water for satisfying users’ quantity and quality requirements. Therefore, China’s water rights trading should pay attention to the water quality and quantity of the exchanged water, and use a unified “Standard Water (SW)” calculation method. This provides a way to convert the amount of water in the transaction based on different water qualities. If the quality of the water rights sold by the transferor is lower than transferee’s minimum requirement, to protect the interests of the transferee, this kind of water rights should be converted into more water as a compensation to the transferee, and also, as a punishment of the transferor. This method can guarantee the environmental justice in water rights trade as much as possible.

Furthermore, the impact of changes in ecosystem services on water value should not be ignored [28]. At present, the studies relating to water and ecological value are of wide concern to scholars [29,30,31,32], and provide theory basis for the measurement of the water ecological value. On considering the degree of water ecological value in different stages of economic development, this paper uses the Pearl Curve Model to express the relationship between the development stage coefficient of environmental ecological value and the level of social and economic development. 

The above methods provide a good reference for the measurement of the practical value of water in this research. However, in the actual water rights transaction, the value of water rights provided by the transferor is not constant for the transferee. When the water rights value changes, it is necessary to use certain standards to convert the water rights, for reward and punishment. Till now, few researches and applications are directly linked to the calculation of Standard Water. Wu et al. [6] proposed that water rights of different quality can be converted. Zhang et al. [33] put forward a water rights trading mode of “converting quantity according to quality” for industrial enterprises. Neither of them gave a specific conversion method. Min et al. [25] and Zhang et al. [34] considered the effect of water quality, establishing an incentive mechanism of rewarding excellence and punishing inferiority for water rights allocation, but this conversion method does not relate to the value of water rights. Some other studies, such as the calculation of water pollution equivalent [35] and standard coal [36] etc., embody the idea of standardization, but they cannot be applied to water rights trading.

Through the analysis above, this paper incorporates water scarcity degree, water quality and water ecological value in a standardization system for measuring the practical value of water resources. From the perspective of rewarding the good and punishing the bad, the standard water conversion model is constructed according to the practical value of water resources, which can improve the existing water rights trading theory and provides a scientific measurement standard for the development of water rights trading. The rest of this paper is arranged as follows: Section 2 puts forward the theoretical interpretation of SW, its measurement method and describes the case study. Section 3 presents the results and discussion. Finally, the conclusions and recommendations are formed in Section 4.

## 2. Methods and Materials

### 2.1. Theoretical Interpretation of SW Measurement

#### 2.1.1. Discourses of Water Scarcity 

Water scarcity is a gradually increasing threat to ecosystems, livelihoods and consensus, and is one of the hot topics of the global crisis. In 1992, the International Conference on Water and the Environment in Dublin mentioned that “taking water as an economic interest to manage is an important way to achieve effective and equitable water utilization and encourage the water conservation and protection” (ICWE, 1992). Hence, it is of great significance to study the real connotation of water scarcity for the analysis of water rights issues. Nowadays, water scarcity is always taken for granted. People tend to pay direct attention to the shortages of water supply caused by natural forces, such as drought, rather than the water use practices and social and political factors caused by human beings. Faced with this phenomenon, some scholars use discourse analysis to analyse deeply water scarcity in different countries or regions. In addition, the water scarcity discourse should be placed in a broader bilateral relationship, including geopolitical developments and intergovernmental interests, in order to understand the different factors that affect water scarcity [37]. For example, Mehta [38] put forward that the water scarcity can be reflected in two aspects of “real” and “manufactured”, that is to say, it is not only a biophysical phenomenon, but also a discourse construction. Hussein [39] identified the elements comprising the discourse of water scarcity in Jordan from water insufficiency and water mismanagement narratives. Edwards [40,41] discussed the significant role of the mismanagement in the Australia neoliberal policy mechanisms. These researches provide a reference for us to understand the constituent factors of water scarcity and explore the necessity for water right transactions.

China, as a great factor in the population of the world, faces more serious water resources crises. Water scarcity is an important factor in the aggravation of water crises. On the basis of Chinese national conditions, population growth and unequal water distribution and other natural factors directly cause water scarcity in most of China, but with the faster economic development, manmade serious waste and pollution of water also aggravates the water scarcity. Although the establishment of water rights and the implementation of a series of water rights management systems alleviate the pressure of Chinese water scarcity, uneven distribution of water rights among regions still exist. Therefore, some regions with scarce water must obtain part of their water rights from water rich regions through water rights trading. It is necessary for us to evaluate the water value provided by the transferor in water rights trades and put forward water right conversion methods with different values.

#### 2.1.2. Environmental Justice in Water Rights Trade

The importance of water and its primacy in many cultures have promoted the establishment of the human rights nature of water, and give the government the obligation to provide people with sufficient water resources [42]. Generally, citizens’ right to water can be defined as the right of the citizens to obtain sufficient, clean water to meet individual needs. The respect, protection and realization of citizens’ water rights need to be regulated from the two perspectives of water supply and water demand. Combined with the theory of environmental justice [43,44], the water resources demand emphasizes environmental distribution justice, while the water supply side attaches more importance to environmental correction justice. Distributive justice is to distribute benefits or burdens according to certain standards, emphasizing the equality of proportion [45]. The distribution justice of water resource demand emphasizes that the water resource allocators need consider the interests of multiple water users, carefully analyze the immediate interests and long-term planning of social and economic development, comprehensively weigh the water demand and characteristics of different regions in the basin, formulate scientific, reasonable and sustainable water resource distribution system, and ensure the fairness and rationality of water resource allocation. Obviously, the core significance of regulating water rights from the perspective of water demand lies in justice and equity. What’s more, the environmental distribution justice generally takes place in the process of “distribution”, while the environmental correction justice takes place in “transaction”. Corrective justice is the remedy for the improper behavior in the equal trade, which pursues the equal quantity and the balance of interests between the two sides of the trade. The corrective justice in the water supply is mainly embodied in emphasizing the fairness and efficiency of water resource transaction, which is also the core significance of regulating water right from the water supply perspective. 

However, it should be noted that the water rights trade is also a way to reallocate water rights between water rich areas and water deficient areas. Hence, both of the environmental collation and correction justice should be considered in the trade. Compared with the water rights transferor (water rich area), the water rights transferee (water shortage area) is more sensitive to the quantity and quality of water right and the economic benefits it can bring. In the water rights pricing and water supply, the water rights transferor is in a natural dominant position, and can often make huge profits by selling at a high price or providing low-quality water. In the absence of a perfect market control mechanism, the water shortage is the weak environmental justice party in the trade. In the long run, this can easily cause social order instability and mass events. Therefore, from the perspective of protecting the interests of water shortage subjects, a fair standard water conversion method should be established to form a trade convention (that’s why we put forward the measurement of Standard water (SW)). It is important to correctly evaluate the value of the target water for the water rights transferee, and convert the target water according to the actual value of the water. In turn, if the actual value of the target water provided by the transferor is higher than the minimum requirement of the transferee, this conversion method can also protect the interests of transferors to some extent.

#### 2.1.3. Theoretical Interpretation of SW Measurement

The essence of SW is to recalculate the exchanged water quantity in the transaction according to its practical value based on a predetermined standard. To measure the practical value of exchanged water, comprehensive factors such as the original value of water resources, water scarcity, water environmental quality and ecological factors are taken into consideration. 

The purpose of SW measurement aims protect the legitimate rights and interests of both parties, motivate the transferor’s aspiration to provide better quality water, and curb the occurrence of shoddy goods. In this way, fair trade is maintained, negotiation cost is reduced, continuity of the transaction process is guaranteed, and the water environment is effectively protected. From the perspective of law, measurement of SW is helpful to reflect the fairness of both sides of the transaction; From the perspective of sociology, measurement of SW is helpful to reduce obstacles of water rights trading, protect the water environment and maintain social harmony and stability.

**Figure 1:**
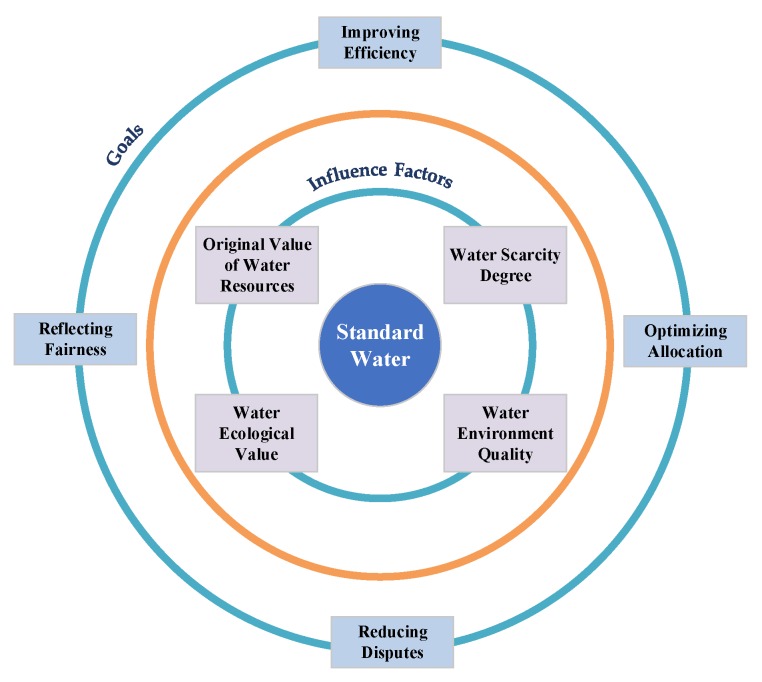
The influence mechanism of SW.

Based on the analysis above, this paper proposes the concept of SW as follows: SW is the standardized water quantity converted from the original quantity of exchanged water required in the contract after considering its original value, scarcity degree, water environment quality, water ecological value, etc. and on the premise of safeguarding the normal rights and interests of both sides in the transaction. The logic frame diagram of the factor decomposition of SW measurement and goals it will realize is seen in Figure 1.

### 2.2. Methods

#### 2.2.1. The Framework of Standard Water Measurement Model

**Figure 2:**
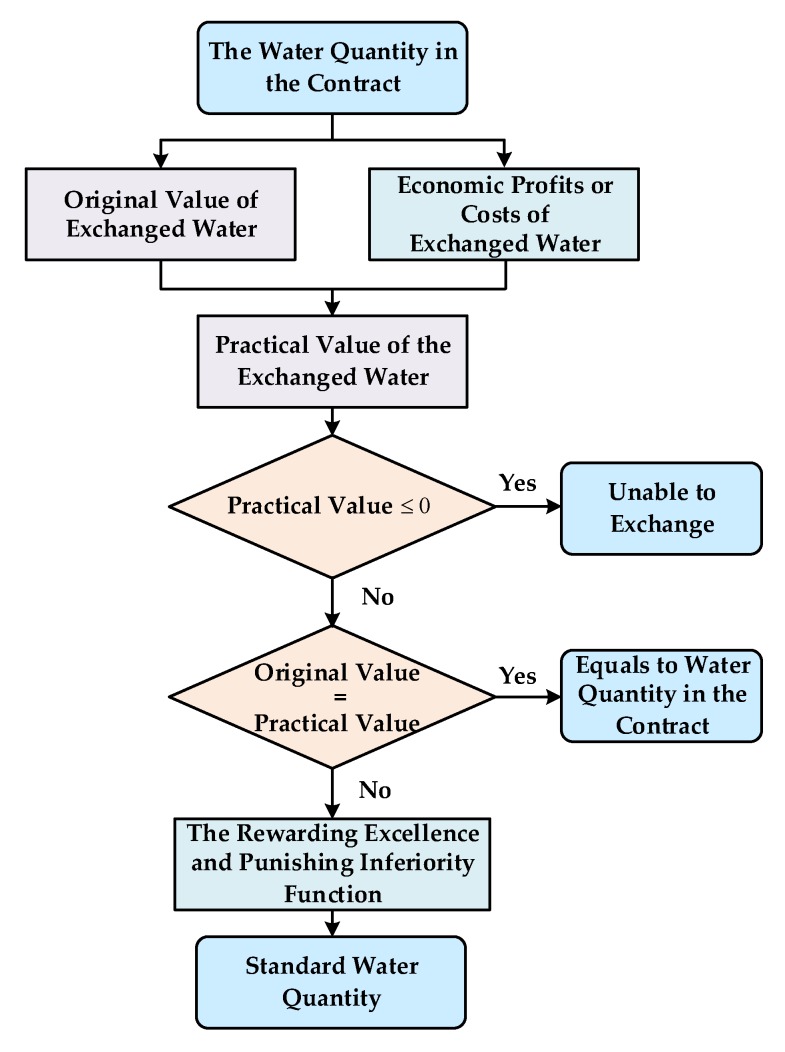
Procedures of Standard Water measurement.

Based on the original value and practical value of water resources, this paper establishes a rewarding excellence and punishing inferiority function on water quality, and sets up a SW measurement model for water rights trading. The measurement mechanism of SW includes three steps. Step 1 is to measure the original value of exchanged water, which can be obtained by the shadow price model based on input-output table; Step 2 is to get the practical value of the exchanged water according to the measurement of economic profits or costs and the original value obtained in Step 1; Step 3 is to establish the SW quantity calculation model according to the rewarding excellence and punishing inferiority function, which is built according to the original value and practical value got in Step 1 and Step 2. If the practical value is negative, the transaction will be meaningless for the transferee. Hence, the trade makes sense only when the practical value is positive. According to the analysis above, we designed the standard water measurement processes as in Figure 2.

#### 2.2.2. The Original Value of the Exchanged Water

The water resources utilization system is a complex giant system composed of economy, society, resources and environment, and its composition determines that the essence of water resources value is a unity of the value of economy, society, resources and environment. In the market economy, price is the ultimate expression of value, so the value of water resources is finally reflected in its price. However, due to the regional differences, the non-uniform water quality, and the differences of the formation and evaluation time for market price, when evaluating the value of water resources according to the market transaction price, a series of corrections are need to be made, such as the regional correction, the quality correction, the transaction situation correction, the time correction, etc. [46]. In addition, because of the long-term or short-term water scarcity, and the monopoly of water resources ownership, the development and utilization of water resources are subject to various constraints and restrictions in various regions, and there is no completely free water resources market in China. It is inevitable that there is a big deviation between the water market price and its practical value. Therefore, using the market price to measure the value of water resources is one-sided and cannot reflect the value of water resources comprehensively and objectively. Based on the shadow price, this paper takes the scarcity of resources as the value basis and the marginal benefit of resources as the value scale to measure the original value of the exchanged water. The purpose is to reflect the marginal contribution of resources to the target value, the marginal value of resources under the optimal decision, the market supply and demand of resources and the degree of scarcity. So that the value of water resources can be reflected objectively by its shadow price.

Shadow price is a kind of theoretical price. Starting from the limitation of resources, it is a quantitative analysis of the use value of resources with the full, reasonable distribution and effective utilization of resources as the core. Therefore, shadow price reflects the value of resources under the condition of market equilibrium. According to the character of shadow price and the input-output relationship between industries, the mathematical model of shadow price of transferee j is established [47,48].

(1) The objective function is:

(1) $$\max Z=\sum_{m=1}^{M}a_{vm}Y_{m}$$

where m=1,2,⋯,M; Z is the total added value of various industries in the national economy; $a_{vm}$ is the value-added coefficient of the *m*^th^ industry, which is equal to the added value of the industry and the ratio of total output; $Y_{m}$ is the final products of the *m*^th^ industry; M is the total number of industries.

(2) The constraints

The constraints of the shadow price mathematical model include two aspects. One is the constraint of production resources, which means social production activities are limited by the amount of water resources; another is the constraint production index, which means social production needs to meet the lower bound constraint of final products, so as to avoid the failure to guarantee residents’ life under the principle of maximizing economic benefits. The constraints of the model are expressed as follows:

(2) $$\{\begin{array}{c} \left(I-A\right)X=Y\\ a_{wm}X_{m}\leq W_{m}\\ \sum_{m=1}^{M}a_{wm}X_{m}\leq W\\ Y^{g}\leq Y\\ X^{g}\leq X\leq X^{h} \end{array}$$

where A is input-output direct consumption coefficient matrix, its component element $a_{nm}$ (n,m=1,2,⋯,M) equals the ratio of the product value of the *m*^th^ output sector consumed in the production of the *n*^th^ input sector to the total output of the *m*^th^ input sector; Y is the final product column vector; $a_{wm}$ is the direct water use coefficient of the *m*^th^ industry, which is equal to the ratio of the total water consumption and the total output of the industry; $X_{m}$ is the total output of the *m*^th^ industry; W is the total amount of water resources available; $W_{m}$ is water resources available of the *m*^th^ industry; $Y^{g}$ is lower bound column vector for final product; $X^{g}$ is the total output lower bound column vectors; $X_{h}$ is the total output of the upper bound column vectors; *g* and *h* are symbols that reflect the lower bound and upper bound.

Equations (1) and (2) aim to maximize the sum of added values of the sectors contained in the model, when it is constrained by the input-output balance, upper and lower limit constraints of total output, water quantity that actually available. The model can be solved by LINGO, and after that, the shadow price $P_{Shadow}$ of water resource can be obtained. $P_{Shadow}$ can be subdivided into different industries, such as agriculture water shadow price $P_{Shadow-A}$, industry water shadow price $P_{Shadow-I}$, domestic water shadow price $P_{Shadow-D}$, and ecological water shadow price $P_{Shadow-E}$. According to the shadow price of the transferee’s water resources and the water quantity in the contract $W_{Contract}$, the original value of the exchanged water $I_{j(Original)}$ to the transferee j can be get, is can be expressed as Equation (3):

(3) $$I_{j(Original)}=P_{Shadow}\cdot W_{Contract}$$

#### 2.2.3. The Economic Benefits or Costs of the Exchanged Water

Due to the fact that the emissions of pollutants into rivers and lakes that must be strictly controlled in different water rights trading regions are not single, for example, the main pollutant control indexes of the seven major river basins in China include COD, NH_3_-N and TP, etc., the pollutant control index of over proof discharge that needs to be considered is not singular, but multiple. In fact, when measuring the “value” of tradable water rights of transferor and transferee of water rights, it is necessary to comprehensively consider the different superposition effects of multiple pollutants in water resources of both sides on their water environment. This paper measures the economic benefits or costs of the exchanged water, which is used to reflect the economic profits or losses of the actual trading water to the transferee relative to the exchanged water quality required in the contract. To measure the impact of water pollution on the relationship between economic activities [49,50], this paper establishes the economic benefits or costs model using Equation (4):

(4) $$C_{j(PL)}=\sum_{f=1}^{5}lS_{W}P_{j}W_{Contract}\lambda_{f}-\sum_{f=1}^{5}lS_{W}P_{j}W_{Contract}\lambda_{f(Actual)}$$

where $C_{j(PL)}$ means the economic benefits or costs of the exchanged water, when the actual water quality is better than that specified in the contract, $C_{j(PL)}>0$, otherwise, $C_{j(PL)}<0$. The parameters of Equation (4) are as follows:

(1) l is the water ecological value development stage coefficient. For the development stage coefficient of water ecological value will change with the development level of social economy, the relationship between them can be expressed by Pearl R growth curve model [51] behind:

(5) $$l=1/(1+e^{3-1/E})$$

where *E* means the Engel coefficient, and reflects the transferee’s affluence.

(2) $S_{W}$ is the water scarcity index. $S_{W}$ can be measured by the ratio of water demand to water supply, the more abundant the water resource is, the less its scarcity value is, and vice versa:

(6) $$S_{W}=\frac{W_{demand}}{W_{supply}}$$

(3) $\lambda_{f}$ is the water environment function loss rate of different water quality classes. $\lambda_{f\left(Actual\right)}$ is the water environment function loss rate of an actual water quality class. At present, China’s surface water environmental quality standards divides surface water quality into five categories, and water bodies inferior to Class V basically have no functional use. This paper defines water quality class f=1,2,3,4,5, referring to water quality Class I, II, III, IV and V. According to the thought of Xiao and Mao [49], the environmental function loss rate of different water quality classes, $\lambda_{f}$ is set as shown in Table 1.

(4) Other parameters. $P_{j}$ means the water price of waterworks in transferee’s region, $W_{Contract}$ means the water quantity specified in the contract.

#### 2.2.4. Standard Water Measurement Model by Rewarding Excellence and Punishing Inferiority

The quality, scarcity and the impact on the ecological environment of the exchanged water is different. By using the cost-benefit method, the model is given by Equation (7):

(7) $$I_{j(Practical)}=I_{j(Original)}+C_{j}{}_{\left(PL\right)}$$

where $I_{j(Practical)}$ means the practical value that the transferee may generate by purchasing, and the practical benefit is equal to the total original value of the exchanged water plus the economic benefits or costs. When $I_{j(Practical)}\leq 0$, $I_{j(Original)}\leq -C_{j}{}_{\left(PL\right)}$, the cost of water rights transaction equals or greater than the original value that can be obtained for the transferee, and the transaction is meaningless. Hence, water rights transactions can only be carried out when it meets Equation (8):

(8) $$I_{j(Practical)}>0$$

In the process of a water rights transaction, it is helpful to judge the rationality of the transfer price to convert the target exchanged water of different values into the SW quantity according to the equivalent conversion method. This paper uses the ratio of the original value to the practical value to show the gap between the original value and the practical value. In fact, the original value refers to the economic value of the water provided by the transferor to the transferee without considering other influence factors; and the practical value reflects the economic value of water resources on considering the impact of water scarcity, water quality and other factors, the impact can be positive or negative. When $\frac{I_{j\left(Original\right)}}{I_{j\left(Practical\right)}}\in (0,1]$, it means the practical value of the exchanged water is higher than (or equal to) its original value, and it applies to the rewarding function; When $\frac{I_{j\left(Original\right)}}{I_{j\left(Practical\right)}}\in (1,+\infty )$, it means the practical value of the exchanged water is lower than its original value, it applies to the punishing function. The rewarding excellence and punishing inferiority function are set with the following rules.

First, the rewarding function is a monotone decreasing function between (0,1], and it ranges from [0,+∞). Second, the punishing function is monotone increment function between (1,+∞), and it ranges from (0,1). Third, when the quality of water reaches a certain level, there will be no significant change in its marginal utility when the quality of water keeps increasing, so the reward function should set a highest value.

**Figure 3:**
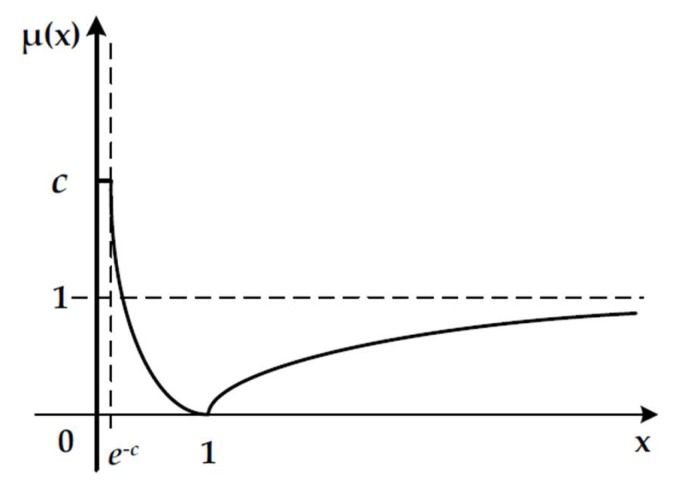
The rewarding excellence and punishing inferiority function.

Referring the thought of Zhang and Wu [37] and the analysis above, this paper establishes the rewarding excellence and punishing inferiority function as given by Equation (9) and Figure 3.

(9) $$\mu (x)=\{\begin{array}{ccc} c & , & 0<x\leq e^{-c},c>0\\ -\ln x & , & e^{-c}<x\leq 1\\ \frac{2}{\pi }\arctan(x-1) & , & x>1 \end{array}$$

where $x=\frac{I_{j\left(Original\right)}}{I_{j\left(Practical\right)}}$. As Figure 3 shows, when x∈(0,1], μ(x) is the rewarding coefficient; when x∈(1,+∞), μ(x) is the punishing coefficient.

To reflect that when the quality of water reaches a certain high level, it will have little effect for the transferee’s usage, this paper sets a highest value of the rewarding coefficient c, c>0. The certain value of c can be determined by the two parts in different transactions on considering the detailed usage of water resources and other matters. The higher c means a higher incentives mechanism, and the transferee needs water with higher practical value. The sensitivity analysis of the different values c is shown in Appendix A.1.

With the rewarding excellence and punishing inferiority function (9), the SW measurement model of water rights trading is constructed as in Equation (10):

(10) $$W_{S}=\{\begin{array}{cc} W_{Contract}\left[1+\mu \left(\frac{I_{j\left(Original\right)}}{I_{j\left(Practical\right)}}\right)\right] & ,I_{j(Practical)}>I_{j(Original)}\\ W_{Contract} & ,I_{j(Practical)}=I_{j(Original)}\\ W_{Contract}\left[1-\mu \left(\frac{I_{j\left(Original\right)}}{I_{j\left(Practical\right)}}\right)\right] & ,I_{j(Practical)}<I_{j(Original)} \end{array}$$

where $W_{s}$ means SW.

### 2.3. Case Study

#### 2.3.1. Case Description

Dongyang City and Yiwu City, adjacent to each other, are located in the Jinhua-Quzhou Basin in central Zhejiang Province, southeast China. Both of them belong to the Qiantang River Basin and are located in the upper reaches of Jinhua River, an important tributary of the Qiantang River. Compared with Yiwu City, Dongyang City is richer in water resources. Besides the Nanjiang river and other abundant streams, there are two large reservoirs in Dongyang City, the Hengjin Reservoir and Nanjiang Reservoir. For Yiwu City, though it is a famous commodity production base in China with a high GDP, the shortage of water resources seriously restricts its sustainable economic and social development. The socio-economic and water resources status of the two cities in 2000 is summarized in Table 2.

**Figure 4:**
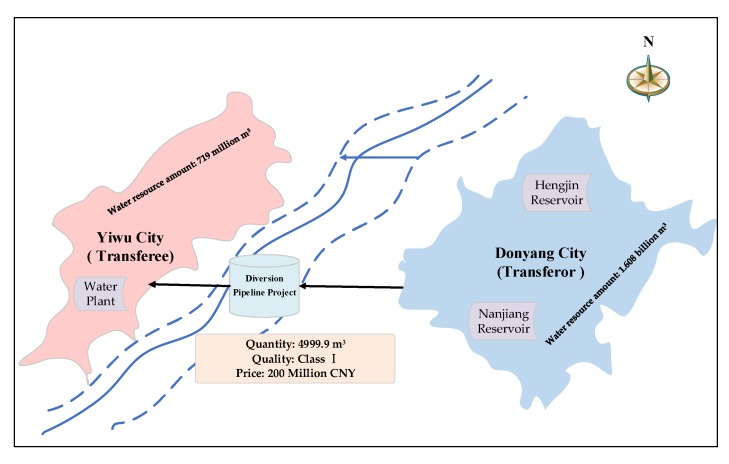
Schematic diagram of the Dongyang-Yiwu water rights transaction.

Due to the drought, Yiwu City has suffered from water crises several times, but the adjacent Dongyang City has relatively abundant water resources. In 1995 and 1996, under the coordination of higher government, Dongyang City provided more than 2 million m^3^ of high-quality water resources to Yiwu City twice. In order to take a long-term view, Yiwu City put forward the idea of trans-regional water diversion from Dongyang City. After negotiation, the two cities formed a water rights transfer agreement in Dongyang City on November 24th, 2000. The core content of the water rights transaction contract is that Yiwu City would invest 200 million CNY to purchase the permanent right to use 49.999 million m^3^ water resources of Class I quality from the Hengjin Reservoir. A schematic diagram of the transaction is shown in Figure 4.

The Dongyang-Yiwu water rights transaction is of great significance to the establishment of China’s water rights trading market. Firstly, it set a precedent for China’s regional water rights trading. Secondly, it forced the Chinese government to introduce water rights trading policies. Thirdly, it provided a reference for improving the efficiency of water resources utilization. However, due to the fact it is the first water rights transaction practice carried out in China, both parties of the transaction lack any experience for reference. In addition, the contract stipulates that “matters not covered herein shall be settled through negotiation by both parties”, in which there also lays a hidden potential for disputes in the future. To reflect the applicability of the model, this paper stipulates the exchanged water quality is in Class II to Class V rather than in Class I as the contract requires, and measures its SW.

#### 2.3.2. Data Collection and Sources

The related data on social situation and development status of Dongyang City and Yiwu City are sourced from Jinhua Statistical Yearbook (1996-2001), Zhejiang Statistical Yearbook (2001), and Jinhua Statistical Bulletin on National Economic and Social Development (2000). The water resources data on water situation of the two cities are from the Jinhua Water Resources Bulletin (2000) and Zhejiang Water Resources Bulletin (2000) and Yangtze River Basin & Southwest Rivers Water Resources Bulletin (2000). 

## 3. Results and Discussion

### 3.1. The Original Value of the Exchanged Water

Based on the shadow price measurement model of equations (1) and (2), the shadow price of the transferee Yiwu City can be obtained. Due to the similar situation of Yiwu City and the whole Yangtze River Basin, the calculation process of shadow price can be referenced according to Liu and Zou [47]. The process of shadow price calculation is shown in Appendix A.2. For the usage of the exchanged water, this paper uses the industry shadow price to obtain the original value. Using the procedures given in Appendix A.2, the shadow price of the Yiwu City in 2000 is 4.02 CNY/m^3^. For the water quantity in the contract $W_{Contract}$ of 49.999 million m^3^, with Equation (3), the original value of water resources in this case can be obtained, $I_{j\left(Original\right)}$ is 200.996 million CNY.

As the original value of water resources is the value of the water itself, and the embodiment of its owner in economy. For industrial, agricultural and domestic water use, the direct value of water resources to users is the marginal value of the product [52]. In the process of water rights trading, taking the original value of the exchanged water into account expresses the importance of water resource of the transferee, and helps to reflect the transferee’s willingness of payment. 

### 3.2. The Practical Value of the Exchanged Water

For the water ecological value development stage coefficient l, according to the statistical yearbook and water resources bulletin of Yiwu City and Zhejiang Province, the Engel coefficient *E* of Yiwu City was 28.3% in 2000, so according to Equation (5), l is 0.631.

For the water scarcity index $S_{W}$, by the water resources bulletin, the average water demand of Yiwu City in 2000 was 150,000 m^3^/d, and the average supply was 90,000 m^3^/d. Hence, according to Equation (6) the water scarcity index $S_{W}$ was 1.667.

Combined with revenue data of waterworks, the water price of waterworks at that time was 1.5 CNY/m^3^. According to Equation (4) and Table 1, when f=1,2,3,4,5, the corresponding $C_{j(PL)}$ will be obtained, and with Equation (7), the corresponding $I_{j(Practical)}$ will obtained as is Table 3.

The measurement of practical value is a reflection of the relative scarcity and ecological value of water resources. The scarcity of resources is one of the fundamental problems that the water rights can be exchanged and an important factor must be considered in commodity exchange [53,54]. Areas with higher relative scarcity have higher willingness to purchase water rights. Moreover, water resources in the same quality have higher utility for water-deficient areas. On the premise of not damaging the local residents’ right to normal use of water, the convert quantity of water resources in relatively scarce areas should be appropriately increased. The ecological value of water resources can not only reflect the environmental quality of water resources, but also reflect the availability of water resources [55]. Due to the different environmental conditions of water pollution in different places, the water quality of the exchanged water is also different. Therefore, when the water resources usufructs are considered as a commodity, its “quality” must be considered. In addition, water rights transaction promotes the conversion of water resources between the transferor and transferee. The increase or decrease of water resources as well as the development and utilization of water resources have a certain impact on the ecosystem of the region where both parties are located. To reflect such impact in the transaction process, the water ecological value generated by the underlying transaction water needs to be measured.

In a certain year, the water ecological value development stage coefficient and water scarcity index of the transferee are determined values, the economic profits or costs are related to the water quality. In this case, if the water Dongyang City provided is in Class II-V, it will cause economic costs from 7.891 to 59.179 million CNY, so the practical value of the exchanged water will be decreased from 193.105 to 141.817 million CNY. The results show that practical value will be obviously changed when the water quality is different from the contract. According to Equation (4), when the transactions are executed during different periods, the social, economic and water scarcity situation will also have changed, so the practical value will change accordingly.

### 3.3. Standard Water Quantity

According to Equation (9) and Table 3, with the $I_{j}{}_{\left(Original\right)}$ obtained in 3.1, which is 200.996 million CNY, the ratio between the original value to the practical value is shown in Table 4.

Table 4 shows that $\frac{I_{j\left(Original\right)}}{I_{j\left(Practical\right)}}>1$, which means μ is the punishing coefficient in this case. Combined the calculation above and Equation (10), the corresponding SW in different water classes will be obtained, as listed in Table 5.

The rewarding or punishing coefficient is related to the original value and practical value of the exchanged water. In this case, when the quality of exchanged water becomes worse, the quantity will be reduced as a punishment. When the exchanged water provided by Dongyang City is in Class II-V, the punishment coefficient will be 0.026-0.252, and the corresponding SW will be reduced to 48.699-37.399 million m^3^. In addition, the practical value is also related water ecological value development stage, water scarcity and the water price of waterworks, when the transaction executed in different periods, the corresponding SW will also be different, which means we need to measure SW dynamically during the execution period of the contract.

## 4. Conclusions and Recommendations

### 4.1. Conclusions

Water rights transactions are an effective market means to promote water resource conservation and optimal allocation. However, there are also market failures in the water rights market (such as negative externalities caused by the change of water use, water quality not meeting the production requirements of the transferee, etc.), which require the water administrative department to implement strong supervision on the wrong behaviors of the main body in the whole process of transaction. Most of the existing literatures on water rights trading is based on an ideal situation, that is, both sides of water rights trading can restrict their own behavior and abide by the provisions of the contract. However, in the process of the execution of the actual transaction contract, a breach of contract by one or both parties still exist. Even though we know that we need to use reward and punishment mechanism to restrain this phenomenon, there are few studies on quantitative analysis of reward and punishment amounts. Therefore, it is important to consider the impact of the transferor’s transaction behavior on the transferee in water rights trade, and quantitatively analyze the reward or punishment to the transferor.

This paper proposes the concept of SW, and establishes the measurement model through the perspective of rewarding excellence and punishing inferiority. In this model, we evaluate the original and practical value of water rights in the trade. Then we focus on the gap between the original and practical value of water produced by the transferor to the transferee in trades, and modify the original value through the development stage coefficient of water ecological value, water resource scarcity index, water quality, etc. Finally, we take China’s first water rights practice, the Dongyang-Yiwu water rights transaction, as a case study. In this case, Dongyang City is required to transfer 49.999 million m^3^ water of Class I quality to Yiwu City. If the water Dongyang City provides is in Class II-V, according to the SW measurement model of this paper, the water quantity will be converted to 48.699-37.399 million m^3^. Compared to the contract, the exchanged water quantity decreased by 1.3-12.6 million m^3^, which reflects the view of punishment, and the results show this method is effective and applicable.

The measurement of SW will help to promote the environmental justice in water rights trade, avoid possible disputes caused by different water quality, and protect the rights and interests of vulnerable groups in the transaction. SW measurement in water rights trading has high practicability and applicability to ensure the fairness of transaction, improve the efficiency of transaction and reduce the subsequent transaction disputes. The method is generally suitable for the current three water rights trading modes in China (regional water rights trading, water access trading, irrigation water user trading).

### 4.2. Recommendations

China’s water rights trading market is developing at a high speed, and laws and regulations are being continuously established and improved. Hoverer, the market is still far from being a mature water market, both the operation and guarantee systems need to be perfected. In order to ensure the long-term and orderly development of water rights trading, this paper puts forward the following suggestions:

(1) Specifying the detailed requirements for multiple properties of the exchanged water in water rights transactions. Comprehensively consider the practical value of the exchanged water before transactions, and then formulate scientific and reasonable trading strategies, so as to ensure the fairness and rationality of the water right trading, and maximize the interests of both parties.
(2) Establishing dynamic water quantity or transaction pricing mechanism for water rights trading. In view that the current long-term contracts have fixed water quantity and price, to divide the total execution period into several stages, and measure SW at the end of each stage as an assessment. The dynamic trading water quantity or price is then adjusted according to the assessment results.
(3) Improving the guarantee system for water rights trading. The government and the water rights trading platforms should constantly improve and optimize the guarantee system of water rights trading, supervise and guide each link of water rights trading, and accelerate the establishment of a mature market-oriented and government-supervised water rights trading system.

Due to the limitations of data and models, this study did not take factors into consideration such as time and engineering fees. In the future, we will consider the influence of exchange costs and engineering costs to improve the standardization model, and make it more suitable for long-term water rights contracts. What’s more, the SW method is more of an empirical model, and some parameters of standard water measurement models are based on our experience in water resource management, which need to be further demonstrated in the future through group decision-making or other methods. More influence factors can be tried in the model in the future, such as effect of geopolitical relations.

## Figures and Tables

**Table 1:** Environmental functional loss rate of different water quality classes.

Water Quality Class	I	II	III	IV	V
$\lambda_{f}$	0	0.10	0.25	0.50	0.75

**Table 2:** The socio-economic and water resources status of Dongyang City and Yiwu City in 2000.

Index	Dongyang City	Yiwu City
**Land Area (km^2^)**	1739	1103
**Population (thousand people)**	788	668
**Cultivated area (ha)**	25004	22912
**Per capita GDP (CNY)**	12794	17945
**Annual average water resources (million m^3^)**	1608	719
**Per capita water resources (m^3^)**	2126	1130

**Table 3:** The practical value of the exchanged water in different water quality classes (million CNY)

Water Quality Class	I	II	III	IV	V
$C_{j(PL)}$	0	−7.891	−19.726	−39.453	−59.179
$I_{j(Practical)}$	200.996	193.105	181.270	161.543	141.817

**Table 4:** Ratio original value to the practical value of different water quality class.

Water Quality Class	I	II	III	IV	V
**Ratio**	1	1.041	1.109	1.244	1.417

**Table 5:** SW quantity in different water quality class (million m^3^).

Water Quality Class	I	II	III	IV	V
μ	0	0.026	0.069	0.152	0.252
$W_{s}$	49.999	48.699	46.549	42.399	37.399

**Figure A1:**
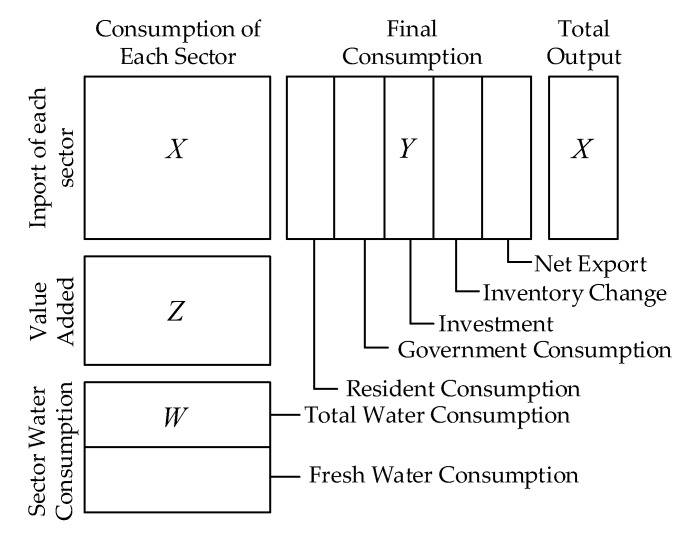
Concept diagram of water input and output table.

## Appendix A

### Appendix A.1. The Sensitivity Analysis of Different Highest Rewarding Function Value

According to the rewarding excellence and punishing inferiority function (9) and the SW measurement model (10), corresponding quantity of $W_{S}$ with different highest rewarding function value c of different values are shown in Table A1.

**Table A1:** Corresponding SW with the different highest value of the rewarding function.

c	0.5	1.0	1.5	2.0	2.5	3.0	3.5	…	+∞
$W_{S}$	$1.5W_{contract}$	$2W_{contract}$	$2.5W_{contract}$	$3W_{contract}$	$3.5W_{contract}$	$4W_{contract}$	$4.5W_{contract}$	…	+∞

With Table A1, the administration can determine the highest rewarding function value c. With the increase of c, the original amount of water is converted into more and more SW, indicating that the reward is more and more powerful. The practical value of water rights provided by transferor is higher than original value for the transferee. That is to say, the value of water has increased, per unit of water in the contract worthies more after being rewarded. For example, when c is 0.5, 1 m^3^ of water in contact is worth 1.5 m^3^.

### Appendix A.2. The Caculation Processes of the Shadow Price

According to the existing studies [47,56,57,58], the calculation of the shadow price of water resources includes classifying the sectors, formulating the water consumption input and output table and then according to Equations (1) and (2), using the statistical analysis software such as LINGO to get the detailed shadow price of different industries. Of the 51 sectors, the sectors 1 to 39 are non-water conservancy sectors and sectors 40 to 51 are water conservancy sectors, of which names and classifications are given in Table A2.

**Table A2:** Names and classifications of the 51 sectors.

Non-water Conservancy Sectors		Water Conservancy Sectors
01. Agriculture 02. Coal mining and processing 03. Crude petroleum and natural gas products 04. Metal ore mining 05. Non-ferrous mineral mining 06. Manufacture of food products and tobacco processing 07. Textiles 08. Clothing, leather, furs, down and related products 09. Sawmills and furniture 10. Paper and products, printing and recording medium production 11. Petroleum processing and coking 12. Chemicals 13. Nonmetal mineral products 14. Metal smelting and pressing 15. Metal products 16. Machinery and equipment 17. Transport equipment 18. Electric equipment and machinery 19. Electronic and telecommunications equipment 20. Instruments, meters, cultural and office machinery	21. Maintenance and repair of machine and equipment 22. Other manufacturing products 23. Scrap and waste 24. Electricity, steam, and hot-water production and supply 25. Gas production and supply 26. Construction 27. Freight transport and warehousing 28. Post and telecommunications 29. Wholesale and retail trade 30. Dining and drinking places 31. Passenger transport 32. Finance and insurance 33. Real estate 34. Social services 35. Health services, sports, and social welfare 36. Education, culture and arts, radio, film, and television 37. Scientific research 38. General technical services 39. Public administration and other sectors	40. Construction of flood and drought control 41. Management of flood and drought control 42. Construction of ecological water environment protection 43. Ecological water environment protection (non-construction) 44. Wastewater treatment 45. Construction of water supply and comprehensive use projects 46. Management of water supply and comprehensive use projects 47. Agriculture and rural household water supply 48. Urban and industrial water supply 49. Hydroelectric power 50. River transport 51. Freshwater fish farming

The concept diagram of water input and output table [57] is as seen in Figure A1, and the water consumption input and output table can be formulated as in Table A3.

**Table A3:** Structure of water consumption input and output table.

	Indirect Water Consumption				Final Water Consumption	Total Output
	Agriculture	Industry	Domestic and Service	Ecology		
Agriculture	$X_{11}$	$X_{12}$	$X_{13}$	$X_{14}$	$Y_{1}$	$X_{1}$
Industry	$X_{21}$	$X_{22}$	$X_{23}$	$X_{24}$	$Y_{2}$	$X_{2}$
Domestic	$X_{31}$	$X_{32}$	$X_{33}$	$X_{34}$	$Y_{3}$	$X_{3}$
Ecology	$X_{41}$	$X_{42}$	$X_{43}$	$X_{44}$	$Y_{4}$	$X_{4}$
Value Added Coefficient	$a_{v1}$	$a_{v2}$	$a_{v3}$	$a_{v4}$		
Total Water Consumption	$W_{1}$	$W_{2}$	$W_{3}$	$W_{4}$		

According to the analysis above and the water resources input-output table, the water usage coefficient can be obtained as in Table A4 [56], and the shadow of each industry can be obtained with equations (1) and (2) by LINGO. The shadow price is shown in Table A5.

**Table A4:** The direct water usage coefficient table of the 51 sectors.

**Sector No.**	01	02	03	04	05	06	07	08
**Direct Water Usage Coefficient**	0.1353	0.0039	0.0076	0.0070	0.0063	0.0086	0.0052	0.0048
**Sector No.**	09	10	…	41	42	43	44	45
**Direct Water Usage Coefficient**	0.0041	0.0301	…	0.0007	0.0012	0.0159	0.0012	0.0016
**Sector No.**	46	47	48	49	50	51		
**Direct Water Usage Coefficient**	0.0035	0.0047	0.0048	0.0005	0.0021	0.1732		

**Table A5:** The shadow prices of different industries (CNY)

Industry	Agriculture	Industry	Domestic	Ecological
**Shadow Price**	1.07	4.02	5.26	1.62
